# Factors Associated with Glomerular Yield in Percutaneous Kidney Biopsy

**DOI:** 10.3390/jcm12123877

**Published:** 2023-06-06

**Authors:** Kenta Torigoe, Ryosuke Sakamoto, Shinichi Abe, Kumiko Muta, Hiroshi Mukae, Tomoya Nishino

**Affiliations:** 1 Department of Nephrology, Nagasaki University Hospital, 1-7-1 Sakamoto, Nagasaki City 852-8501, Nagasaki, Japan; s-ryosuke@nagasaki-u.ac.jp (R.S.); s-abe@nagasaki-u.ac.jp (S.A.); k-io@nagasaki-u.ac.jp (K.M.); tnishino@nagasaki-u.ac.jp (T.N.); 2 Department of Respiratory Medicine, Nagasaki University Graduate School of Biomedical Sciences, 1-7-1 Sakamoto, Nagasaki City 852-8501, Nagasaki, Japan; hmukae@nagasaki-u.ac.jp

**Keywords:** kidney biopsy, kidney disease, glomerular yield, procedural adequacy, pathological diagnosis, anemia

## Abstract

Percutaneous kidney biopsy is essential for diagnosing various kidney diseases. However, insufficient glomerular yield leads to misdiagnosis, a critical problem. We retrospectively investigated the risk of insufficient glomerular yield in percutaneous kidney biopsies. We included 236 patients who underwent percutaneous kidney biopsies between April 2017 and September 2020. We retrospectively analyzed the relationship between glomerular yield and patient characteristics. After the biopsy, 31 patients produced insufficient glomerular yields (cases with yielded glomeruli <10). Glomerular yield correlated negatively with hypertension (β = −0.13, *p* = 0.04), and positively with glomerular density (β = 0.59, *p* < 0.0001) and the volume of the biopsy core (number of punctures, number of biopsy cores, total length of biopsy core, length of core collected by one puncture, and cortical length). Patients yielding <10 glomeruli had lower glomerular densities (14.4 ± 1.6 vs. 22.9 ± 0.6/cm; *p* < 0.0001). These results suggest that glomerular density is crucial to glomerular yield. Furthermore, glomerular density was negatively correlated with hypertension, diabetes, and age. Hypertension was independently associated with low glomerular density (β = −0.16, *p* = 0.02). Thus, the glomerular yield was associated with glomerular density and biopsy core length, and hypertension might be related to glomerular yield via low glomerular density.

## 1. Introduction

Percutaneous kidney biopsy is essential for diagnosing various kidney diseases. Kidney biopsy is usually considered in cases of glomerular hematuria, proteinuria, or rapidly progressive glomerulonephritis [1]. Pathological diagnosis via kidney biopsy provides more information for treatment decisions. In addition, kidney biopsy improves kidney prognosis in patients with CKD (chronic kidney disease) and kidney dysfunction, especially in cases of severe proteinuria [2]. Since Iversen and Brun’s first kidney biopsy report in 1951 [3], the procedure has developed continuously. Currently, the ultrasound-guided percutaneous kidney biopsy is widely used and is safer than a blind kidney biopsy [4]. The major complication of a kidney biopsy is post-biopsy bleeding; however, insufficient glomerular yield is also a critical problem. Glomerular yield indicates the diagnostic power of kidney biopsy for kidney diseases, and the biopsy core must include at least 10 glomeruli for optimal diagnosis [5,6]. Inadequate sampling can sometimes mislead clinical judgment; therefore, it is imperative to collect sufficient tissue samples to avoid an insufficient glomerular yield. However, in recent years, the proportion of insufficient glomerular yield in percutaneous kidney biopsy has increased in the United States, and the reason for this trend is speculated to involve changes in operator specialty and patient aging [7]. Therefore, assessing the risks of insufficient glomerular yields is essential for optimal diagnosis and to avoid misleading clinical judgment. A previous study revealed that old age and lower serum albumin levels were associated with fewer collected glomeruli in percutaneous kidney biopsies [8]. However, few studies focus on the risk factors of insufficient glomerular yield in percutaneous kidney biopsy. Therefore, to address this gap, this study retrospectively investigated the risk factors associated with insufficient glomerular yield in ultrasound-guided percutaneous kidney biopsies.

## 2. Materials and Methods

### 2.1. Patient Selection

In total, 236 patients who underwent percutaneous kidney biopsies at the Department of Nephrology, Nagasaki University Hospital, between April 2017 and September 2020, were retrospectively analyzed. Patients who underwent percutaneous kidney biopsies for kidney transplantation and those without data on glomerular yield were excluded from the study.

### 2.2. Data Collection

Data measured before the kidney biopsy constituted the baseline patient data. If red blood cell transfusion was performed before the kidney biopsy, the expected increase in hemoglobin (Hb) after transfusion (dividing the dosed hemoglobin amount [g] by the circulating plasma volume [dL]) was added to the Hb level before the kidney biopsy. Six patients underwent blood transfusions before kidney biopsy due to anemia. A nephrologist performed kidney biopsies using a 16 G needle under ultrasonic guidance. Details of the procedure used in our hospital have been previously described [9,10]. The core samples were divided for assessment using light microscopy (LM), immunofluorescence microscopy (IF), and electron microscopy (EM). However, only a minimal length of cores was used for IF and EM, compared with LM. Therefore, the number of glomeruli collected via kidney biopsy was obtained from the LM pathology report. The lengths of the obtained cores were measured via LM using a specimen slide. Glomerular density was calculated by dividing the number of sample glomeruli by the cortex length.

### 2.3. Statistical Analysis

Categorical variables were expressed as numbers (%), continuous variables as mean ± standard deviation, and non-normally distributed data as medians and interquartile ranges. Simple and multiple linear regression analyses were performed to investigate factors associated with glomerular yield. Insufficient glomerular yield was defined as collected glomeruli of <10 based on a previous study [5,6], and we compared patients with sufficient and insufficient yield. We compared nominal variables between both groups using Fisher’s exact or Pearson’s chi-squared test, while the Wilcoxon rank-sum *t*-Test was used to compare continuous variables. Simple and multiple linear regression analyses were performed to investigate factors associated with glomerular density. These factors were compared between patients with a post-biopsy Hb decrease >1 g/dL and those with ≦1 g/dL, based on a previous study to investigate whether factors associated with glomerular yield were risk factors for post-biopsy bleeding [11]. Statistical analyses were performed using the JMP version 16 software (SAS Institute Inc., Cary, NC, USA). Statistical significance was set at *p* < 0.05.

## 3. Results

### 3.1. Patient Characteristics

Patient characteristics are presented in Table 1. The mean age was 54.4 ± 18.2 years, and 126 (53.4%) were men. The mean pre-biopsy eGFR was 54.9 ± 28.0 mL/min/1.73 m^2^. Through kidney biopsy, 1.18 ± 0.49 cm of a biopsy core was obtained, and the core yielded 20.5 ± 11.2 glomeruli. Only 13% of cases yielded <10 glomeruli. Post-biopsy bleeding was determined using the mean post-biopsy Hb decline, which was 0.29 ± 0.82 g/dL, and 16% of cases had a post-biopsy Hb decline of >1 g/dL. The histopathological diagnosis of IgA nephropathy was the most common (76 patients, 32.2%), followed by anti-neutrophil cytoplasmic antibody-associated glomerulonephritis in 20 patients (8.5%), lupus nephritis in 18 (7.6%), and membranous glomerulonephritis with 18 patients (7.6%). One patient (0.4%) was difficult to diagnose due to a low number of glomeruli (Supplemental Table S1).

### 3.2. Factors Associated with Insufficient Glomerular Yield

Table 2 presents the correlation between glomerular yield and patient characteristics. Yield was negatively correlated with hypertension (β = −0.13, *p* = 0.04), and positively with both glomerular density (β = 0.59, *p* < 0.0001) and factors associated with the volume of the biopsy core recovered (number of punctures, number of biopsy cores, total length of biopsy core, lengths of core collected by one puncture, and cortical length). Multiple linear regression analysis revealed that glomerular density (β = 0.71, *p* < 0.0001) and cortical length (β = 0.76, *p* < 0.0001) were independently positively correlated. At least 10 glomerular samples are required for the optimal diagnosis of kidney pathology; thus, patient characteristics were compared between patients yielding <10 glomeruli and those yielding more.

As presented in Table 3, patients yielding <10 glomeruli had lower numbers of punctures (2 (1 to 3) vs. 3 (2 to 3); *p* = 0.0024) and biopsy cores (2 (1 to 2) vs. 2 (2 to 3); *p* < 0.0001). Additionally, shorter lengths of total biopsy core (0.75 ± 0.08 cm vs. 1.24 ± 0.03 cm, *p* < 0.0001), shorter lengths of core collected by one puncture (0.54 ± 0.04 cm vs. 0.73 ± 0.02 cm, *p* = 0.0002), and shorter lengths of cortex in the biopsy core (0.53 ± 0.07 cm vs. 1.04 ± 0.03 cm; *p* < 0.0001) were observed. Furthermore, glomerular density was low in patients yielding <10 glomeruli (14.4 ± 1.6/cm vs. 22.9 ± 0.6/cm; *p* < 0.0001). Hypertension was common in patients yielding <10 glomeruli; however, the difference was not significant (64.5% vs. 47.8%; *p* = 0.08). Other patient characteristics and laboratory data were similar between both groups. Post-biopsy Hb decline and the prevalence of a post-biopsy Hb decline >1 g/dL were also similar between both groups. Factors related to biopsy core volume and, thus, to the procedure were associated with glomerular yield. The yield was also associated with low glomerular density—a patient-related factor. We next investigated factors related to glomerular density.

As presented in Table 4, older age (β = −0.18, *p* = 0.007), hypertension (β = −0.21, *p* = 0.001), and diabetes mellitus (β = −0.16, *p* = 0.0169) were negatively correlated with glomerular density. In particular, hypertension displayed an independent negative correlation with glomerular density (β = −0.16, *p* = 0.02). As indicated in Table 2, hypertension was also negatively correlated with glomerular yield; however, age and diabetes mellitus were not, suggesting that hypertension is related to glomerular yield via low glomerular density.

### 3.3. Relationship between Biopsy Glomerular Yield and Post-Biopsy Bleeding

We examined the relationship between post-kidney biopsy bleeding and factors related to glomerular yield to investigate the association between the quality and safety of kidney biopsy. We compared factors related to biopsy yield in patients with a post-biopsy Hb decline of >1 g/dL to those with ≦1 g/dL. As presented in Table 5, the glomerular yield was similar in both groups. Factors related to glomerular yield were also similar in both groups. However, the longest core collected in one puncture was longer in patients with Hb decreases of >1 g/dL than in those with ≦1 g/dL (0.79 ± 0.04 cm vs. 0.69 ± 0.02 cm; *p* = 0.03). These results suggest that the high quality and safety of kidney biopsies are compatible. However, collecting longer core in one puncture could be a risk factor for post-biopsy bleeding.

## 4. Discussion

Glomerular yield in percutaneous kidney biopsy was associated with the glomerular density and total length of the biopsy core, especially cortical total length. No association existed between glomerular yield and clinical characteristics; however, hypertension was negatively associated with glomerular density. Furthermore, the length of the longest core collected in one puncture was associated with Hb decline post-biopsy. Conversely, post-biopsy Hb decline was not associated with glomerular yield or the total length of the biopsy core.

Previous reports have revealed that biopsy length correlates with the number of glomeruli [8,12,13]. In this study, similar to previous studies, the number of glomeruli was related to the total length of the core and related biopsy parameters (number of punctures, length of the longest core collected in one puncture, and length of cortical tissue). Glomeruli exist mainly in the kidney cortex [14]. In this study, the number of glomeruli sampled was unassociated with the length of the medulla.

Few studies of percutaneous kidney biopsy focus on the relationship between patient characteristics and glomerular yield; however, a previous study reported a negative correlation between age and glomerular yield [8]. The mechanism of this relationship was speculated to be the age-dependent decrease in the number of nephrons in the kidney [15]. Moreover, the resulting cortical atrophy renders the puncture procedure challenging in aged patients [7]. Consistent with a previous study, age was negatively correlated with glomerular density. Glomerular density was independently positively correlated with glomerular yield, and low glomerular density was a risk factor for yields of <10 glomeruli. Hypertension and diabetes were also factors associated with glomerular density and may be risk factors for insufficient glomerular yield. Older age, hypertension, and diabetes were not risk factors for patients yielding <10 glomeruli in this study. Nonetheless, hypertension was negatively correlated with glomerular yield in the univariate analysis. Therefore, hypertension may be a risk factor for inadequate glomerular yield via low glomerular density. Low birth weight is reportedly associated with low glomerular density [16,17] and might be a risk factor for inadequate glomerular yield. However, information on birth weight was unavailable in this study. Low glomerular density may be influenced by the factors described above, and the overlap of various factors, such as hypertension, older age, and diabetes, may contribute to the risk of the yields of <10 glomeruli. Operator specialization (such as nephrologist or radiologist), imaging modality (ultrasound-guided or computed tomography), and the gauge of the biopsy needle are reportedly associated with glomerular yield [8,11,18,19,20]. In this study, a nephrologist performed all kidney biopsies using a 16 G needle under ultrasonic guidance. Therefore, these factors did not affect the study results.

Obtaining the adequate number of glomeruli without serious bleeding complications is essential. In this study, the number of collected glomeruli and the length of the collected core were similar between patients with post-biopsy Hb declines of >1 g/dL and ≦1 g/dL. However, the length of the core collected in a single puncture was longer in patients with post-biopsy Hb declines of >1 g/dL. Particularly, deeper punctures and larger amounts of core collected simultaneously are associated with the risk of bleeding after a kidney biopsy. The kidney medulla contains large blood vessels [7]; thus, a deeper puncture could injure these vessels, causing a larger Hb decline after the biopsy. Previous studies have reported that it is possible to collect a sufficient number of glomeruli while reducing hemorrhagic complications by using an appropriate puncture depth calculated from height and weight [21]. However, since the depth of puncture was estimated from the length of the core in this study; therefore, the possibility of error with the true depth of puncture is a limitation of this result.

This study has several other limitations. First, this was a retrospective observational study, and the existence of unrecognized confounding factors that affect the observed associations cannot be excluded. Second, kidney biopsy requires skill and proficiency; therefore, the glomerular yield might vary with the operator. However, our previous study revealed that the safety and adequacy of kidney biopsies were similar between nephrology trainees and certified nephrologists [9]. Moreover, the glomerular yield was calculated using the biopsy cores for LM. However, the cores were divided into LM, IF, and EM cores. Despite using only minimal core length for IF and EM, the absence of the glomeruli examined using IF and EM might have affected the results. Finally, patients transfused before a kidney biopsy may have had less elevated Hb levels than expected due to intravascular hemolysis, affecting their baseline Hb levels. However, only six patients were transfused before the kidney biopsy. Therefore, the impact is likely small.

In conclusion, glomerular yield in percutaneous kidney biopsy was associated with glomerular density and biopsy core length rather than with patient characteristics. However, hypertension might be a risk factor for inadequate glomerular yield due to low glomerular density. Deeper punctures and the collection of longer cores in single biopsies are associated with a large Hb decline post-biopsy. Therefore, it is important to ensure the safety and adequacy of kidney biopsy by puncturing at an appropriate depth and concurrently avoiding the collection of long cores.

## Figures and Tables

**Table 1:** Patient characteristics.

Characteristic	All Kidney Biopsies (*n* = 236)
Age (years)	54.4 ± 18.2
Male (%)	53.4
AKI (%)	6.0
Hypertension (%)	50
Diabetes mellitus (%)	14.4
Height (cm)	161.7 ± 8.6
Body weight (kg)	59.7 ± 13.9
BMI (kg/m^2^)	22.7 ± 4.4
Systolic BP (mmHg)	128.2 ± 18.6
Diastolic BP (mmHg)	77.2 ± 13.1
Hb (g/dL)	12.2 ± 2.2
Plt (×10^4^/μL)	25.8 ± 9.8
PT-INR	1.00 ± 0.11
APTT (s)	28.6 ± 6.8
CRP (mg/dL)	0.11 (0.04 to 0.47)
Alb (g/dL)	3.4 ± 0.9
BUN (mg/dL)	18 (13 to 25)
Cr (mg/dL)	1.03 (0.77 to 1.55)
eGFR (mL/min/1.73 m^2^)	54.9 ± 28.0
Urinary protein (g/g Cr)	1.38 (0.54 to 4.16)
Length of biopsy kidney (cm)	9.9 ± 1.0
Number of punctures	3 (2 to 3)
Number of glomeruli	20.5 ± 11.2
Number of glomeruli < 10 (%)	13
Number of biopsy cores	2 (2 to 3)
Total length of biopsy core (cm)	1.18 ± 0.49
Length of longest core collected in one puncture (cm)	0.70 ± 0.27
Total length of cortex in biopsy core (cm)	0.97 ± 0.43
Total length of the medulla in biopsy core (cm)	0.09 (0 to 0.32)
Glomerular density (/cm)	21.7 ± 9.1
Post-biopsy Hb decline (g/dL)	0.29 ± 0.82
Post-biopsy Hb decline > 1 g/dL (%)	16

Continuous variables are presented as mean ± standard deviation. Categorical variables are presented as percentages or numbers. AKI, acute kidney injury; Alb, albumin; APTT, activated partial thromboplastin time; BMI, body mass index; BP, blood pressure; BUN, blood urea nitrogen; Cr, creatinine; CRP, C-reactive protein; eGFR, estimated glomerular filtration rate; Hb, hemoglobin; K, potassium; Na, sodium; Plt, platelet; PT-INR, prothrombin time international normalized ratio.

**Table 2:** Relationship between glomerular yield and patient characteristics.

Characteristic	Univariate Analysis				Multivariate Analysis			
	B	95% CI	β	*p*-Value	B	95% CI	β	*p*-Value
Age	−0.07	−0.14 to 0.01	−0.11	0.1				
Male	0.5	−0.93 to 1.94	0.05	0.49				
AKI	−2.57	−5.58 to 0.45	−0.11	0.09				
Hypertension	−1.44	−2.87 to −0.02	−0.13	0.04	0.02	−0.44 to 0.48	0.002	0.92
Diabetes mellitus	−0.71	−2.75 to 1.32	−0.05	0.49				
Height	0.09	−0.10 to 0.24	0.05	0.42				
Body weight	0.02	−0.08 to 0.13	0.03	0.64				
BMI	0.03	−0.30 to 0.36	0.01	0.87				
Systolic BP	−0.05	−0.12 to 0.03	−0.08	0.24				
Diastolic BP	−0.03	−0.13 to 0.08	−0.03	0.63				
Hb	0.31	−0.31 to 0.93	0.06	0.33				
Plt	0.11	−0.03 to 0.26	0.1	0.13				
PT-INR	−6.3	−18.86 to 6.26	−0.06	0.32				
APTT	0.09	−0.13 to 0.30	0.05	0.43				
CRP	−0.01	−0.50 to 0.48	−0.002	0.97				
Alb	−0.9	−2.45 to 0.64	−0.08	0.25				
BUN	−0.07	−0.16 to 0.02	−0.09	0.15				
Cr	−0.63	−1.71 to 0.45	−0.07	0.25				
eGFR	0.04	−0.01 to 0.09	0.11	0.1				
Urinary protein	0.05	−0.28 to 0.39	0.02	0.75				
Length of biopsy kidney	−0.17	−1.61 to 1.27	−0.02	0.81				
Number of punctures	4.68	2.70 to 6.66	0.31	<0.0001				
Number of biopsy cores	5.11	3.35 to 6.87	0.35	<0.0001				
Total length of biopsy core	12.41	9.95 to 14.89	0.54	<0.0001				
Length of longest core collected in one puncture	16.14	11.13 to 21.15	0.38	<0.0001				
Total length of cortex in biopsy core	16.83	14.26 to 19.40	0.65	<0.0001	19.91	18.84 to 20.98	0.76	<0.0001
Total length of the medulla in biopsy core	−1.15	−6.18 to 3.89	−0.03	0.65				
Glomerular density	0.72	0.59 to 0.85	0.59	<0.0001	0.87	0.81 to 0.92	0.71	<0.0001
Post-biopsy Hb decline	−0.49	−2.24 to 1.26	−0.04	0.58				

AKI, acute kidney injury; Alb, albumin; APTT, activated partial thromboplastin time; B, unstandardized regression coefficient; β, standardized regression coefficient; BMI, body mass index; BP, blood pressure; BUN, blood urea nitrogen; Cr, creatinine; CI, confidence interval; CRP, C-reactive protein; eGFR, estimated glomerular filtration rate; Hb, hemoglobin; K, potassium; Na, sodium; Plt, platelet; PT-INR, prothrombin time international normalized ratio.

**Table 3:** Comparison of patient characteristics between patients with insufficient glomerular yield and those with sufficient yield.

Characteristic	Collected Glomeruli < 10 (*n* = 31)	Collected Glomeruli ≥ 10 (*n* = 205)	*p*-Value
Age (years)	56.6 ± 3.3	54.1 ± 1.3	0.47
Male (%)	45.2	54.6	0.34
AKI (%)	6.5	5.9	>0.99
Hypertension (%)	64.5	47.8	0.08
Diabetes mellitus (%)	19.4	13.7	0.40
Height (cm)	161.8 ± 1.6	161.6 ± 0.6	0.90
Body weight (kg)	57.6 ± 2.5	60.0 ± 1.0	0.37
BMI (kg/m^2^)	21.9 ± 0.8	22.8 ± 0.3	0.25
Systolic BP (mmHg)	130.4 ± 3.3	127.9 ± 1.3	0.48
Diastolic BP (mmHg)	78.4 ± 2.3	77.1 ± 0.9	0.60
Hb (g/dL)	12.1 ± 0.4	12.2 ± 0.2	0.84
Plt (×10^4^/μL)	24.0 ± 1.8	26.1 ± 0.7	0.25
PT-INR	1.00 ± 0.02	1.00 ± 0.01	0.90
APTT (s)	27.8 ± 1.2	28.7 ± 0.5	0.51
CRP (mg/dL)	0.08 (0.03 to 0.22)	0.12 (0.04 to 0.50)	0.32
Alb (g/dL)	3.4 ± 0.2	3.4 ± 0.1	0.96
BUN (mg/dL)	20 (15 to 28)	17 (13 to 25)	0.10
Cr (mg/dL)	1.05 (0.80 to 1.77)	1.02 (0.77 to 1.54)	0.54
eGFR (mL/min/1.73 m^2^)	49.9 ± 5.0	55.5 ± 2.0	0.30
Urinary protein (g/g Cr)	3.30 ± 0.77	3.18 ± 0.30	0.88
Length of biopsy kidney (cm)	10.1 ± 0.2	9.9 ± 0.1	0.36
Number of punctures	2 (1 to 3)	3 (2 to 3)	0.0024
Number of biopsy cores	2 (1 to 2)	2 (2 to 3)	<0.0001
Total length of biopsy core (cm)	0.75 ± 0.08	1.24 ± 0.03	<0.0001
Length of longest core collected in one puncture (cm)	0.54 ± 0.04	0.73 ± 0.02	0.0002
Total length of cortex in biopsy core (cm)	0.53 ± 0.07	1.04 ± 0.03	<0.0001
Total length of the medulla in biopsy core (cm)	0.10 (0 to 0.32)	0.08 (0 to 0.34)	0.66
Glomerular density (/cm)	14.4 ± 1.6	22.9 ± 0.6	<0.0001
Post-biopsy Hb decline (g/dL)	0.40 ± 0.15	0.28 ± 0.06	0.45
Post-biopsy Hb decline > 1 g/dL (%)	19.4	15.1	0.60

Continuous variables are presented as mean ± standard deviation. Categorical variables are presented as percentages or numbers. The *p*-values were statistically analyzed using Student’s *t*-test and the Mann–Whitney U-test for continuous variables, and a chi-square test for categorical variables. AKI, acute kidney injury; Alb, albumin; APTT, activated partial thromboplastin time; BMI, body mass index; BP, blood pressure; BUN, blood urea nitrogen; Cr, creatinine; CRP, C-reactive protein; eGFR, estimated glomerular filtration rate; Hb, hemoglobin; K, potassium; Na, sodium; Plt, platelet; PT-INR, prothrombin time international normalized ratio.

**Table 4:** Relationship between glomerular density and patient characteristics.

Characteristic	Univariate Analysis				Multivariate Analysis			
	B	95% CI	β	*p*-Value	B	95% CI	β	*p*-Value
Age	−0.09	−0.15 to −0.02	−0.18	0.007	−0.05	−0.12 to 0.02	−0.1	0.14
Male	−0.31	−1.49 to 0.86	−0.03	0.6				
AKI	−0.85	−3.42 to 1.71	−0.04	0.51				
Hypertension	−1.94	−3.10 to −0.79	−0.21	0.001	−1.43	−2.66 to −0.20	−0.16	0.02
Diabetes mellitus	−2.01	−3.66 to −0.37	−0.16	0.0169	−1.47	−3.12 to 0.17	−0.11	0.08
Height	−0.003	−0.14 to 0.14	−0.003	0.96				
Body weight	−0.04	−0.13 to 0.04	−0.07	0.31				
BMI	−0.17	−0.44 to 0.09	−0.08	0.2				
Systolic BP	−0.02	−0.08 to 0.04	−0.04	0.56				
Diastolic BP	0.008	−0.08 to 0.10	0.01	0.86				
Hb	−0.06	−0.57 to 0.45	−0.01	0.82				
Plt	−0.01	−0.13 to 0.11	−0.01	0.88				
PT-INR	−6.88	−17.21 to 3.46	−0.09	0.19				
APTT	0.12	−0.05 to 0.29	0.1	0.18				
CRP	−0.24	−0.65 to 0.17	−0.08	0.24				
Alb	0.83	−0.44 to 2.10	0.08	0.2				
BUN	0.05	−0.02 to 0.13	0.09	0.18				
Cr	0.32	−0.57 to 1.21	0.05	0.48				
eGFR	0.02	−0.02 to 0.06	0.07	0.29				
Urinary protein	−0.18	−0.46 to 0.09	−0.09	0.19				
Length of biopsy kidney	−0.72	−1.91 to 0.48	−0.08	0.23				

AKI, acute kidney injury; Alb, albumin; APTT, activated partial thromboplastin time; B, unstandardized regression coefficient; β, standardized regression coefficient; BMI, body mass index; BP, blood pressure; BUN, blood urea nitrogen; Cr, creatinine; CI, confidence interval; CRP, C-reactive protein; eGFR, estimated glomerular filtration rate; Hb, hemoglobin; K, potassium; Na, sodium; Plt, platelet; PT-INR, prothrombin time international normalized ratio.

**Table 5:** Comparison between patients with post-biopsy Hb decrease of >1 g/dL and those with ≦1 g/dL.

Characteristic	Hb Decrease > 1 g/dL (*n* = 37)	Hb Decrease ≤ 1 g/dL (*n* = 199)	*p*-Value
Number of punctures	2.5 (2 to 3)	3 (2 to 3)	0.34
Number of glomeruli	21.0 ± 1.8	20.5 ± 0.8	0.79
Number of glomeruli <10 (%)	13	16	0.60
Number of biopsy cores	2 (2 to 3)	2 (2 to 3)	0.91
Total length of biopsy core (cm)	1.26 ± 0.08	1.16 ± 0.03	0.29
Length of longest core collected in one puncture (cm)	0.79 ± 0.04	0.69 ± 0.02	0.03
Total length of cortex in biopsy core (cm)	1.02 ± 0.07	0.96 ± 0.03	0.46
Total length of the medulla in biopsy core (cm)	0.05 (0 to 0.36)	0.09 (0 to 0.30)	0.80
Glomerular density (/cm)	21.4 ± 1.5	21.8 ± 0.6	0.81

Continuous variables are presented as the mean ± standard deviation. Categorical variables are presented as percentages or numbers. The *p*-values were statistically analyzed using Student’s *t*-test and the Mann–Whitney U-test for continuous variables, and the chi-square test for categorical variables.

## Data Availability

The data presented in this study are available on request from the corresponding author.

## Supplementary Materials

The following supporting information can be downloaded at: https://www.mdpi.com/article/10.3390/jcm12123877/s1, Table S1: The pathological diagnosis of patients.
