# Supplementary material for: Factors Associated with Glomerular Yield in Percutaneous Kidney Biopsy
Source: J Clin Med. 2023 Jun 6;12(12):3877. doi: 10.3390/jcm12123877 (PMC10299488; doi:10.3390/jcm12123877)
Supplement: Supplementary file 1 [file jcm-12-03877-s001.zip › jcm-2437703-supplementary.pdf]

Supplemental Table S1. The pathological diagnosis of patients

**Diagnosis**	***n* (%)**
IgA nephropathy	76 (32.2%)
ANCA-associated glomerulonephritis	20 (8.5%)
Lupus nephritis	18 (7.6%)
Membranous glomerulonephritis	18 (7.6%)
Interstitial nephritis	13 (5.5%)
Benign nephrosclerosis	13 (5.5%)
Minor glomerular abnormalities	11 (4.7%)
IgA vasculitis	10 (4.2%)
Minimal change disease	9 (3.8%)
Diabetic nephropathy	8 (3.4%)
Renal amyloidosis	6 (2.5%)
Non-IgA mesangial proliferative glomerulonephritis	5 (2.1%)
Focal segmental glomerulosclerosis	5 (2.1%)
Membranoproliferative glomerulonephritis	4 (1.7%)
Thrombotic microangiopathy	4 (1.7%)
Infection-related glomerulonephritis	3 (1.3%)
Malignant nephrosclerosis	2 (0.8%)
Anti-glomerular basement membrane nephritis	1 (0.4%)
Impossibility of diagnosis	1 (0.4%)
Others	9 (3.8%)
Total	236 (100%)

ANCA, anti-neutrophil cytoplasmic antibody; IgA, immunoglobulin A.
